# Optical Study of Lysozyme Molecules in Aqueous Solutions after Exposure to Laser-Induced Breakdown

**DOI:** 10.3390/biom12111613

**Published:** 2022-11-01

**Authors:** Ruslan M. Sarimov, Tatiana A. Matveyeva, Vera A. Mozhaeva, Aleksandra I. Kuleshova, Anastasia A. Ignatova, Alexander V. Simakin

**Affiliations:** 1 Prokhorov General Physics Institute of the Russian Academy of Sciences (GPI RAS), 119991 Moscow, Russia; 2 Shemyakin-Ovchinnikov Institute of Bioorganic Chemistry, Russian Academy of Sciences, 117997 Moscow, Russia

**Keywords:** laser-induced breakdown, Nd:YAG laser, laser damage of protein, lysozyme activity, lysozyme aggregates, dynamic light scattering, nanoscale objects

## Abstract

The properties of a lysozyme solution under laser-induced breakdown were studied. An optical breakdown under laser action in protein solutions proceeds with high efficiency: the formation of plasma and acoustic oscillations is observed. The concentration of protein molecules has very little effect on the physicochemical characteristics of optical breakdown. After exposure to optical breakdown, changes were observed in the enzymatic activity of lysozyme, absorption and fluorescence spectra, viscosity, and the sizes of molecules and aggregates of lysozyme measured by dynamic light scattering. However, the refractive index of the solution and the Raman spectrum did not change. The appearance of a new fluorescence peak was observed upon excitation at 350 nm and emission at 434 nm at exposure for 30 min. Previously, a peak in this range was associated with the fluorescence of amyloid fibrils. However, neither the ThT assay nor the circular dichroism dispersion confirmed the formation of amyloid fibrils. Probably, under the influence of optical breakdown, a small part of the protein degraded, and a part changed its native state and aggregated, forming functional dimers or “native aggregates”.

## 1. Introduction

The unique ability of the laser to maximize energy concentration in space, time, and spectral range makes this device an indispensable tool in many areas of human activity, and in particular in medicine [1]. There is an intervention in the pathological process or a disease state during the disease’s treatment; this is especially applicable for surgery in the most radical way [2]. Thanks to progress in science and technology, mechanical surgical instruments are being replaced with fundamentally different ones, including lasers [3]. The impact on biological tissues of pulsed laser radiation is determined by a combination of wavelength, energy density, and duration of the radiation pulse. By selecting these parameters, it is possible to vary the biological effects of laser exposure to body tissues. It is possible to separate the thermal and non-thermal effects, for example, by changing the duration of the radiation pulse [4].

Today pulsed lasers with a wide range of pulse duration changes (from milli- to femtoseconds) have come into practice; nanosecond lasers are mostly used. When using nanosecond lasers and lasers with shorter pulse duration, various kinds of nonlinear processes come into play: optical breakdown on the target surface, multiphoton absorption, plasma formation and development, and generation and propagation of shock waves [5]. Evidently, with such a large number of nonlinear processes, it is difficult to create a single laser search algorithm for the desired tasks; in each case, a different approach is required [6]. On the one hand, this is an extremely complicated task; on the other hand, it opened up outstanding possibilities for varying the methods of influencing biological tissue.

One of the most common surgical lasers is the Nd:YAG active medium laser [7]. Such a laser is used for interventions with endoscopic access in pulmonology, gastroenterology, urology, aesthetic cosmetology for hair removal, and interstitial laser coagulation of tumors in oncology [8]. In Q-switched mode with pulse durations from 10 ns, it is used in ophthalmology, for example, in the treatment of glaucoma [9]. Most tissues at the wavelength of the Nd:YAG laser (1064 nm) have a low absorption coefficient [10]. The effective penetration depth of such radiation into tissues can be several millimeters and provides good hemostasis and coagulation [11]. An important advantage of the Nd:YAG laser is the possibility of delivering radiation to the affected area with fiber optic light guides. The use of endoscopic and fiber instruments allows laser radiation to be delivered to the lower and upper gastrointestinal tract in an almost non-invasive way [12].

The development of nonlinear processes in tissue upon absorption of laser radiation underlies the medical action of a laser. One of the main nonlinear processes is an optical breakdown. The optical breakdown is a fast irreversible process of transformation of a medium from transparent to strongly absorbing under the action of intense radiation [13]. Optical breakdown occurs when certain threshold values of laser radiation energy density are exceeded [14]. At energies close to the threshold values, the process of breakdown development is probabilistic [15]. It is known that optical breakdown occurs less frequently the cleaner the irradiated medium is from impurities [16]. Studies of laser breakdown in liquids have shown that the presence of nanosized impurities leads to an increase in the breakdown probability and a decrease in the threshold values of laser radiation energies [17]. The process of optical breakdown of a liquid is much more intense (a few orders of magnitude) in the presence of nanoparticles in the medium [18]. It is not known whether protein molecules, which are essentially nanosized objects, can lead to an increase in the breakdown probability in aqueous solutions. It is also not known what happens to the protein preparation after exposure to optical breakdown. To answer these two questions, a standard Nd:YAG laser with a pulse duration of 10 ns was used in this study.

Many publications study the effect of continuous or pulsed laser radiation in solutions on biomolecules up to the level of optical breakdown. Low-intensity laser radiation is used in many measurement methods and is considered to have no significant effect on the biological object under study. For example, the continuous He-Ne laser is used in this article in the dynamic light scattering method. In addition, laser radiation with non-breakdown intensity is used for a variety of purposes. For example, for initiation of folding using a laser [19], modification of the native state of a protein through the production of hydroxyl radicals using an excimer KrF laser [20], photochemical generation of a burst of hydroxyl radicals for the oxidation of amino acids on the protein surface, which made it possible to study the solvent-accessible areas of the protein surface [21]. In addition, laser flash photolysis is often used to study electron transfer within proteins [22]. Separately, one cannot fail to note the multiphoton absorptions of biomolecules [23], which are possible upon laser exposure.

There are few studies on the effect of laser breakdown on biomolecules. One of the directions in this area is the use of high-power lasers for research in the field of two or multiphoton absorption of biological objects. However, such phenomena are not observed during optical breakdown. For example, in [24], two-photon absorption for DNA was observed at intensities only before optical breakdown.

Another area where a breakdown is used to study biological solutions is the Laser-induced breakdown spectroscopy (LIBS) method. The main limitation of the method is the actual combustion of the sample. Another limitation of the method is that it gives information only about the elemental composition of the protein molecule. Naturally, most proteins have a similar elemental composition; therefore, such works are reduced mainly to the separation of similar protein spectra [25]. However, there are works where laser-induced breakdown spectroscopy is enhanced by nanoparticles (NELIBS), which can improve the analytical performance of LIBS for protein studies [26].

We are not aware of works that would simultaneously study the processes occurring in a solution during optical breakdown and changes in a protein. Hen egg white lysozyme (HEWL) was used as a model protein. HEWL is the most researched protein, and it costs extremely low. It is worth noting that lysozyme became the second protein structure and the first enzyme structure, which was obtained using X-ray crystallography [27], and the first enzyme to contain the complete sequence of all twenty standard amino acids [28].

## 2. Materials and Methods

### 2.1. Laser Exposure

**Figure 1:**
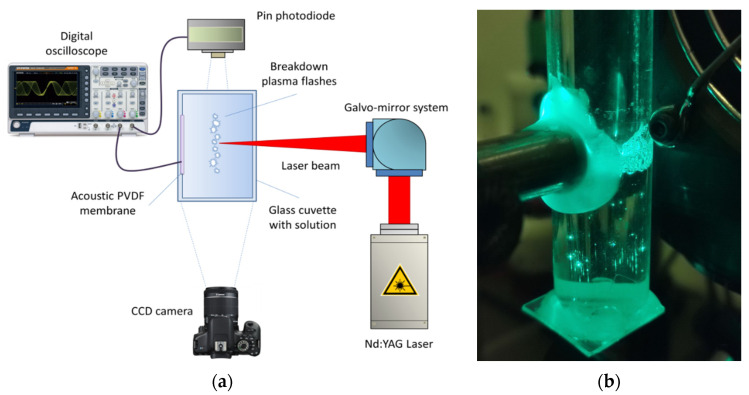
Schematic representation of the experimental setup (**a**). Optical breakdown in the sample with HEWL during laser exposure (**b**).

A schematic representation of the experimental setup is shown in Figure 1a. An Nd:YAG laser (Ekspla NL300, Vilnius, Lithuania) with the following parameters was used as a laser radiation source: pulse duration τ = 4 ns, frequency υ = 1 kHz, wavelength λ = 532 nm, pulse energy ε = 2 mJ. The laser radiation was focused at the center of the cuvette and moved along a straight line 1 cm long at a speed of 500 m/s using a galvanic-mechanical system of mirrors. The movement of radiation in the cuvette is necessary to initiate a breakdown in an unperturbed medium and also to avoid thermal defocusing and additional scattering on the bubbles of the resulting gas. Part of the laser radiation was redirected with a mirror (reflection coefficient 5%) to a pin-photodiode to trigger the time sweep of the oscillograph. The prepared protein solution was placed in a 25 mL glass cuvette. Inside the cuvette—on one of the walls—a piezo-film acoustic sensor was attached in parallel with the scanning line. A pinned photodiode was installed at a distance of 3–4 cm from the cuvette to detect plasma flashes. The signals from the sensor and photodiode were recorded using a GW Instek GDS-72204E digital oscillograph. The plasma flashes were photographed using a Canon EOS 450D digital camera (exposure time 10 ms, ISO 800). For each experimental point, there were at least 50 photographs in one series. Acoustic signals from the sensor and plasma signals from the pin photodiode were analyzed using specially developed LaserCav software (IOFRAN, Moscow, Russia, Version 1.0) (https://drive.google.com/drive/folders/1WQmaSCA4mx2HyRSCxtSiik5MWNku9piR, accessed on 2 September 2022). The plasma images from the camera were analyzed using the LaserImage program (https://drive.google.com/drive/folders/1YRNF2p7qpejlGP55QBiqM108LSGAseaE, accessed on 2 September 2022). The sample volume of the protein solution is 8 mL. The protein concentration is 10 mg/mL. Exposure time up to 30 min. Immediately after exposure, measurements were taken using different methods.

### 2.2. Materials

Hen egg white lysozyme (>20,000 U/mg, A-3711, Applichem), Micrococcus lysodeikticus (lyophilized cells, ATCC No. Aldrich, St. Louis, MS, USA). The water used for the experiments was obtained by distillation and deionization to a resistivity of ~18 MΩ/cm. Dry protein was diluted in clean water and settled during the day to the desired concentration. The protein was dissolved at room temperature using an “IKA RCT digital” (IKA-Werke, Staufen im Breisgau, Deutschland) magnetic stirrer for 30 min at ~5 Hz. The resulting protein solution was used for 48 h.

### 2.3. Absorption Spectra

Absorption spectra were measured on a Cintra 4040 (GBC Cintra 4040, Perth, Australia) in quartz cuvettes with an optical path length of 10 mm at room temperature (~22 °C). The HEWL concentration was 0.4 mg/mL. The absorption spectra were measured with six-eight samples for each group.

### 2.4. Enzyme Assays of HEWL

The activity of HEWL was examined using the lysis of M. lysodeikticus cells at room temperature, as described in [29]. Here, 4 µL of HEWL (0.4 mg/mL) was collected from the initial solution diluted 10 times in water and added, at 100 µL and a concentration of 40 µg/mL, to 2.5 mL of the micrococcus diluted in 20 mM of K2HPO4 (pH = 7.0) to an OD of about 0.7–0.8 (λ = 450 nm). The activity was measured by decreases in OD at the same wavelength with a spectrometer (GBC Cintra 4040, Perth, Australia) for the first two minutes after the addition of lysozyme. Measurements of lysozyme activity were carried out in four samples for each group. The measurements were carried out at room temperature (~22 °C).

### 2.5. Dynamic Light Scattering

Zetasizer ULTRA Red Label (Malvern Panalytical Ltd., Malvern, UK) was used to obtain information on hydrodynamic particle diameters. A 1 mL solution of lysozyme with a concentration of 0.4 mg/mL was measured in a plastic cuvette at 25 °C. Five independent experiments were carried out for the control and for each point of influence. The intensity distributions of the hydrodynamic diameters were calculated using the ZS Xplorer program and algorithm [30].

### 2.6. Fluorescence Spectroscopy

The fluorescence of HEWL in water was studied on a Jasco FP-8300 spectrometer (JASCO Applied Sciences, Victoria, British Columbia, Canada). Measurements of a 1.8 mL solution of lysozyme with a protein concentration of 10 mg/mL were carried out in quartz cuvettes with an optical path length of 10 mm at room temperature (~22 °C). Each sample was measured three times. The figures show typical spectra; with repeated measurements, the intensity maxima change by several percent.

### 2.7. Viscosity Measurement

SmartPave 102 rheometer (Anton Paar GmbH, Graz, Austria) was used to obtain viscosity data of protein solutions. The measuring set was DG26.7 with C-PTD200 cells with 3.8 mL of each sample. All measurements were made at 25 °C, reducing the shear rate from 1000 to 100 s^−1^, using RheoCompass™ software (Anton Paar GmbH, Graz, Austria). The concentration of proteins in the solution was 10 mg/mL.

### 2.8. Raman Spectroscopy

The Raman spectra were recorded on a Senterra II Raman Microscope (Bruker Optik GmbH, Ettlingen, Germany). The spectra were taken from droplets of aqueous solutions of HEWL and BSA dried on a CaF2 substrate at a concentration of 10 mg/mL in the control and after 30 min laser exposure. The device parameters were as follows. Radiation with a wavelength of 532 nm was focused by a 50× objective. The laser power was 12.5 mW, and the accumulation time was 2 s. Averaging over 100 spectra was performed. Each sample was measured at a minimum of 3 points to ensure spectral reproducibility. The obtained spectra were processed by applying (1) concave rubber band correction, (2) Min-Max normalization, and (3) smoothing (number of smoothing points = 17) in the OPUS 8.2.28 program (Bruker Optik GmbH, Ettlingen, Germany).

### 2.9. Refractometry

Refractive index measurements were carried out on a Multiwavelengths Refractometer: Abbemat MW (Anton Paar, Graz, Austria). In the experiments, 1 mL of the solution was poured into the cell of the device, and measurements were made at a wavelength of 435.8, 589.3, and 632.8 nm at a temperature of 25 °C.

### 2.10. Circular Dichroism Spectroscopy

Circular dichroism measurements were carried out on a Jasco J-810 spectropolarimeter. Cuvette was 0.01 cm thick. The protein concentration in the solution during measurements was 1 mg/mL. The measurements were carried out on three independent samples Program used for data calculation: CONTINLL (CDPro package), set of reference spectra: SMP56.

### 2.11. ThT Assay

ThT assay was used to evaluate the amyloid formation. ThT solutions were added to HEWL solution diluted up to 1 mg/mL. The final concentration of ThT was 20 µM. The fluorescence spectra of ThT were recorded between 460 and 560 nm at an excitation wavelength of 450 nm. Assays were carried out in 10 × 10 mm path-length cuvettes without stirring. As a positive control, an 1 mg/mL HEWL solution was placed in 3M GdnHCl and subjected to mixing at 50 °C for 2 or 4 h.

## 3. Results

**Figure 2:**
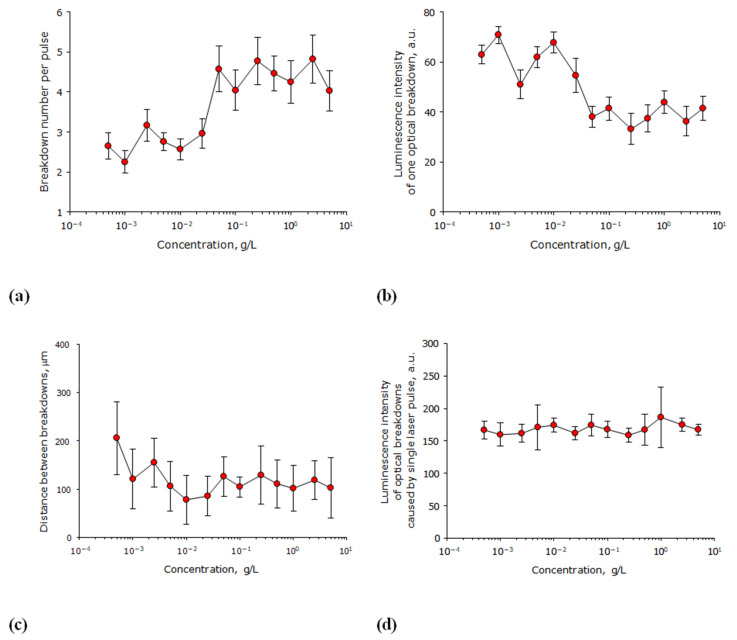
Effect of the concentration of lysozyme molecules in aqueous solution on the characteristics of optical breakdown plasma (*n* = 3, Mean ± SEM). (**a**) Effect of the concentration of lysozyme molecules in an aqueous solution on the number of optical breakdowns caused by a single laser pulse. (**b**) Effect of the concentration of lysozyme molecules in an aqueous solution on the average luminescence intensity of an individual optical breakdown. (**c**) Effect of the concentration of lysozyme molecules in an aqueous solution on the average distance between optical breakdowns caused by a single laser pulse. (**d**) Effect of the concentration of lysozyme molecules in an aqueous solution on the average luminescence intensity of optical breakdowns induced by a single laser pulse.

It is known that one laser pulse, depending on the energy density, can cause in a transparent medium from one to several hundreds of micrometer-sized optical breakdowns. The influence of the concentration of lysozyme molecules in an aqueous solution on the characteristics of the optical breakdown plasma was studied (Figure 2). It has been found that the lysozyme concentration in an aqueous solution can have a significant effect on the number of optical breakdowns caused by a single laser pulse (Figure 2a). At low concentrations of lysozyme up to 0.2 g/L, about 2–3 optical breakdowns are observed per laser pulse. With an increase in the lysozyme concentration to 0.4 g/L, the number of optical breakdowns increases to 4–5 per laser pulse. With further increases in the lysozyme concentration, the number of breakdowns induced with one laser pulse does not change. Usually, with an increase in the number of individual optical breakdowns induced with a single laser pulse, a decrease in the intensity of plasma formation in each optical breakdown is observed. A plasma formation was evaluated by the intensity of luminescence. It has been shown that the lysozyme concentration in an aqueous solution can have a significant effect on the average luminescence intensity of an individual optical breakdown (Figure 2b). At a lysozyme concentration of less than 0.2 g/L, the average luminescence intensity of one laser breakdown is recorded at the level of 50–70 units. With an increase in concentration to 0.5 g/L, the average luminescence intensity of one laser breakdown is steadily recorded at the level of 40 units.

Often in highly scattering media, the distance between optical breakdowns induced by a single laser pulse is connected to the concentration of scatterers. This affects the hydrodynamics observed in the solution and, ultimately, can affect the scenario of the interaction of scatterers, both with each other and with radiation. The influence of the lysozyme molecules concentration in an aqueous solution on the average distance between optical breakdowns caused by a single laser pulse was studied (Figure 2c). It is shown that the distance between individual optical breakdowns of the medium, caused by one laser pulse, at all studied concentrations of lysozyme is approximately 100–200 μm. Statistical differences in the average distances at extreme concentrations (mg/L and g/L) are not observed. However, there is some trend toward a decrease in the distance between breakdowns with increasing protein concentration. An important parameter of the efficiency of plasma formation during the optical breakdown is the luminescence caused by one laser pulse (the sum of the luminescence of all breakdowns caused by one laser pulse); this is an integral estimate of the effectiveness of the interaction of laser radiation with the medium. The effect of the concentration of lysozyme molecules in an aqueous solution on the average luminescence intensity of optical breakdowns induced by a single laser pulse was studied (Figure 2d). It is shown that the average luminescence intensity of optical breakdowns induced by a single laser pulse does not change over the entire range of concentrations studied.

**Figure 3:**
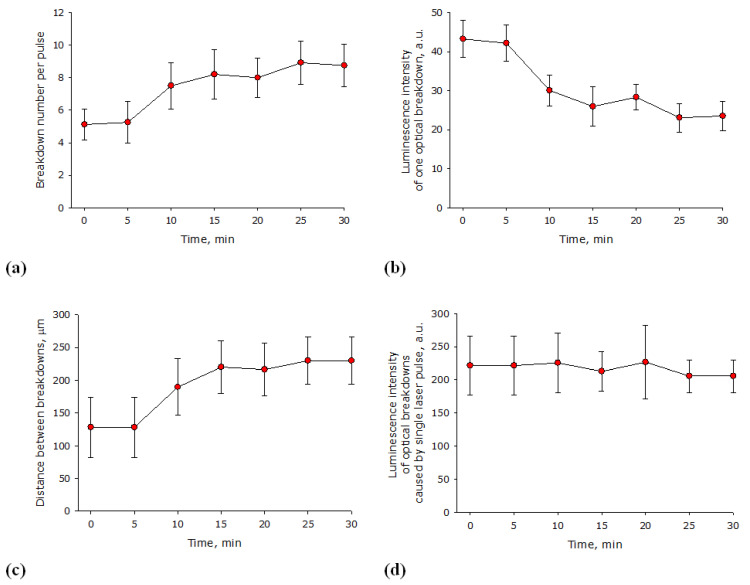
Influence of laser irradiation time of lysozyme aqueous solution (1 mg/mL) on the characteristics of optical breakdown plasma (*n* = 3, Mean ± SEM). (**a**) Effect of laser irradiation time of lysozyme aqueous solution on the number of optical breakdowns caused by a single laser pulse. (**b**) Effect of laser irradiation time of lysozyme aqueous solution on the average luminescence intensity of an individual optical breakdown. (**c**) Effect of laser irradiation time of lysozyme aqueous solution on the average distance between optical breakdowns caused by a single laser pulse. (**d**) Effect of laser irradiation time of lysozyme aqueous solution on the average luminescence intensity of optical breakdowns induced by a single laser pulse.

It is known that the efficiency of the optical breakdown of media varies with the time of exposure to laser radiation. The influence of the irradiation time of lysozyme aqueous solution with a laser on the optical breakdown number induced by a single laser pulse was estimated (Figure 3a). It is shown that optical breakdowns induced by one laser pulse in a lysozyme solution increase from 5 to 8 in 30 min. In general, the process can be described with the equation y = 0.13x + 5.4 (R^2^ = 0.84), where y is the number of breakdowns caused by one laser impulse, x—time in minutes. The influence of the irradiation time of lysozyme aqueous solution with a laser on the average luminescence intensity of an individual optical breakdown was estimated (Figure 3b).

It is shown that after 30 min of laser irradiation, the average luminescence intensity of an individual optical breakdown decreases by almost 25%. In general, the process can be described by the equation y = −0.7x + 42 (R^2^ = 0.82), where y is the average luminescence intensity of an individual optical breakdown, and x is the time in minutes. The influence of the time of irradiation of lysozyme aqueous solution with a laser on the average distance between optical breakdowns caused by a single laser pulse was estimated (Figure 3c). It is shown that the average distance between individual breakdowns increases from 130 µm at the beginning of irradiation to 230 µm after 30 min of laser exposure. The influence of the irradiation time of lysozyme aqueous solution with a laser on the average luminescence intensity of optical breakdowns caused by a single laser pulse was estimated (Figure 3d). It is shown that the average luminescence intensity of optical breakdowns caused by one laser pulse does not change for at least 30 min of exposure to laser radiation on lysozyme aqueous solutions. 

**Figure 4:**
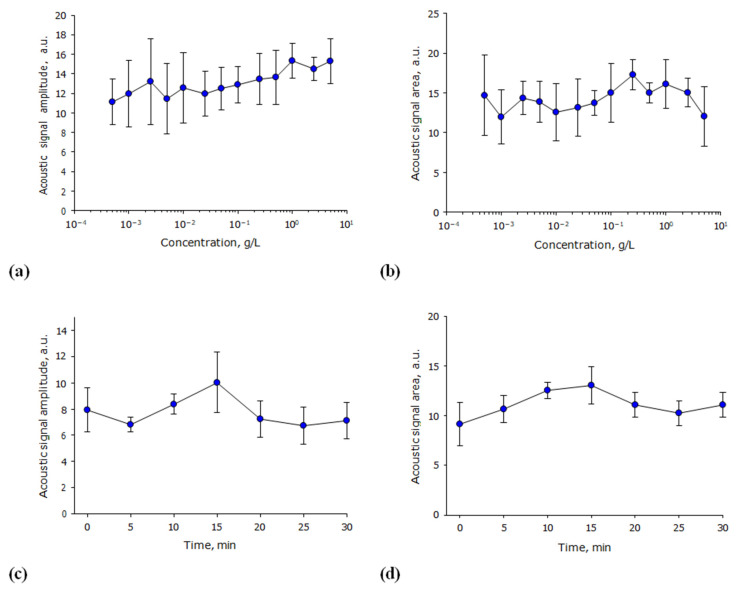
Effect of lysozyme aqueous solution irradiation time and its concentration on the characteristics of acoustic oscillations induced by the optical breakdown (*n* = 3, Mean ± SEM). (**a**) Effect of the concentration of lysozyme molecules in an aqueous solution on the average amplitude of acoustic signals induced by the optical breakdown. (**b**) Effect of the concentration of lysozyme molecules in aqueous solution on the average intensity of acoustic signals induced by the optical breakdown. (**c**) Effect of laser irradiation time of an aqueous solution of lysozyme (1 mg/mL) on the average amplitude of acoustic signals induced by the optical breakdown. (**d**) Effect of laser irradiation time of an aqueous solution of lysozyme (1 mg/mL) on the average intensity of acoustic signals induced by the optical breakdown.

During optical breakdown, microscopic clumps of nonequilibrium plasma appear in the medium, and acoustic oscillations are generated in the elastic medium. The amplitude and average intensity of such acoustic signals are often used to integrally estimate the efficiency of the optical breakdown of a medium. The influence of the concentration of lysozyme molecules in an aqueous solution on the average amplitude of acoustic signals induced by optical breakdown was studied (Figure 4a). It is shown that the average amplitude of acoustic vibrations induced by optical breakdown does not change significantly at all studied protein concentrations; however, with an increase in the lysozyme concentration, a tendency to increase the amplitude of the acoustic vibration is observed. The influence of the lysozyme molecules concentration in an aqueous solution on the average intensity of acoustic signals induced by optical breakdown was estimated (Figure 4b). It has been established that the average intensity of acoustic signals induced by optical breakdown does not change significantly at all studied protein concentrations. The influence of the irradiation time of lysozyme aqueous solution with a laser on the average amplitude and average intensity of acoustic signals induced by optical breakdown was studied (Figure 4c,d). It is shown that the average amplitude and average intensity of acoustic signals induced by optical breakdown do not change significantly during at least 30 min of exposure. 

Thus, the plasma parameters and acoustic oscillations study allow us to state that the optical breakdown efficiency at protein concentrations of more than 0.5 g/L has no significant differences. It follows from this that the main parameters of an optical breakdown do not differ significantly when the protein concentration changes from 0.5 g/L and higher, which means that we have the right to compare the results obtained from irradiating proteins at different concentrations in the range of 0.5–10 g/L. As for the time factor, the physical parameters of optical breakdown change monotonically with time, according to patterns close to linear. 

**Figure 5:**
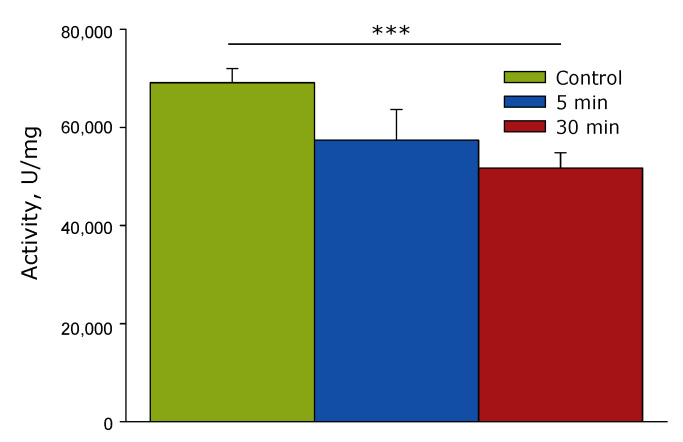
Effect of the laser irradiation time of lysozyme aqueous solution on its catalytic activity (*n* = 4, Mean ± SD). Control is a solution of HEWL before exposure to laser radiation. ***—*p* < 0.001, significance levels for one-way ANOVA.

The influence of the time of laser irradiation of lysozyme aqueous solution on its catalytic activity was studied (Figure 5). It has been established that after the action of laser radiation on lysozyme aqueous solution, a decrease in its catalytic activity is observed. So, when exposed for 30 min, the activity of lysozyme statistically significantly decreases by almost 30%. To find out what caused the decrease in activity, spectral studies of an aqueous solution containing lysozyme were carried out.

**Figure 6:**
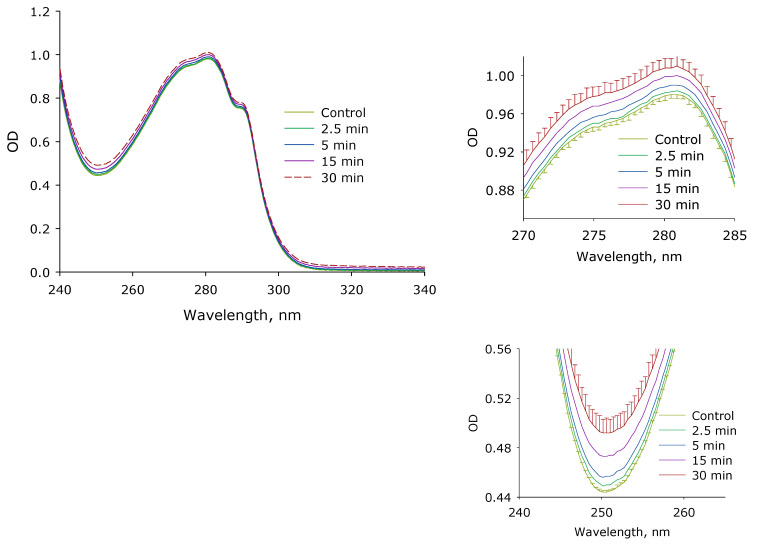
Effect of laser irradiation time on the optical density of lysozyme aqueous solution (*n* = 6–8, Mean ± SD). The upper right panel shows in enlarged form the change of optical density in the region of the local maximum at 280 nm. The lower right inset presents an enlarged view of the change of optical density in the region of the local minimum at 250 nm. The data were obtained using sub-nanometer differential two-beam spectroscopy.

The effect of laser irradiation time on the optical density of an aqueous solution of lysozyme is shown in Figure 6. It is shown that under the action of laser radiation, the optical absorption of a lysozyme solution increases. Moreover, the absorption in the wavelength range of 250–280 nm increases linearly for 30 during exposure to laser radiation. The absorption of protein molecules in aqueous solutions increases for almost 0.05 units, both at the local minimum (252 nm) and the local maximum (280 nm). It should be noted that similar changes also occur in the longer wavelength region (right shoulder of the peak, 310–320 nm). Thus, an increase in the absorption intensity is observed both in the spectral absorption range of aromatic amino acid residues and in the range after 310 nm. Thus, we can assume several options for the development of events. The first option, chemical modification of aromatic amino acid residues, which increases optical absorption, can also be a change in absorption due to partial denaturation of the protein. In addition, the optical density may increase due to non-specific scattering caused by, for example, slight protein aggregation.

**Figure 7:**
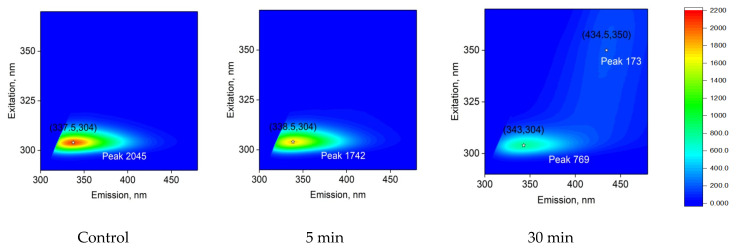
Effect of laser irradiation time on the fluorescence of a protein solution of lysozyme 10 mg/mL (typical spectrum). Three-dimensional fluorescence maps are presented. The abscissa shows the range of emission wavelengths in nm (λem). The ordinate shows the range of excitation wavelengths in nm (λex). The fluorescence intensity is expressed in relative units using a color scale, which is the same for all three spectra. The numbers in parentheses on the graphs indicate the local fluorescence maxima in the coordinates (λex; λem). Fluorescence intensity is indicated with the word Peak and a number.

Figure 7 shows the effect of the time of exposure to laser irradiation on the fluorescence of the lysozyme protein solution. It is shown that the fluorescence excitation maximum is observed at 304 nm and does not change after exposure of the lysozyme solution to laser radiation, both for 5 min and for half an hour. The fluorescence intensity decreases by 15% after 5 min of exposure to laser radiation and by more than 60% when exposed to laser radiation for 30 min. In this case, the maximum emission for an intact protein solution and a solution exposed to laser radiation for 5 min is 337–338 nm. When the protein is irradiated for 30 min, the emission maximum will shift by several nanometers into the “red” region (343 nm). The shape of the fluorescence spot on 3D maps does not change significantly. It should be noted that when the protein solution is irradiated with laser radiation for 30 min, a weak fluorescent spot (maximum 434 nm) appears upon excitation at 350 nm.

Previously, an increase in fluorescence upon excitation by 357 with an emission peak at 430 nm was observed during the formation of amyloid aggregates of lysozyme during protein incubation at 50 °C in an acidic medium (50 mM glycine-HCl buffer, pH 2.2) [31]. Thus, it has been shown that under the action of laser radiation on protein solutions, a decrease in the intensity of fluorescence of aromatic amino acid residues is observed, as well as the formation of amyloidosis structures.

**Figure 8:**
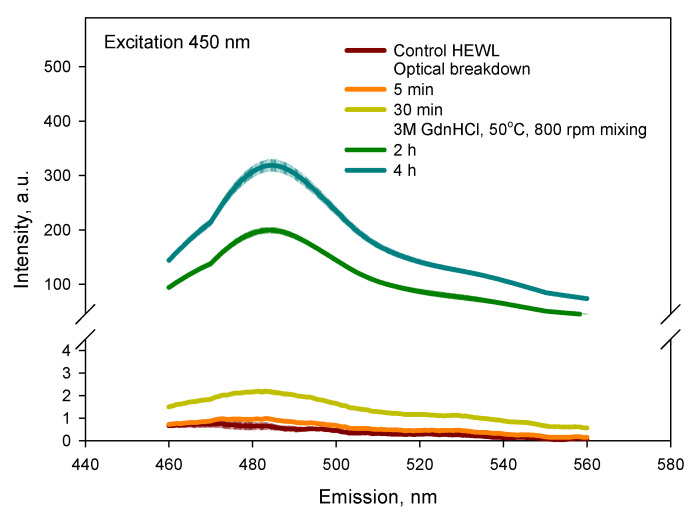
ThT assay for control HEWL solution and after 5 and 30 optical breakdowns during laser exposure. As a positive control, 1 mg/mL HEWL solution was placed in 3M GdnHCl and subjected to mixing at 50 °C for 2 or 4 h.

To check the formation of amyloid fibrils, a ThT assay was performed. Figure 8 shows the fluorescence of the ThT for the HEWL control solution and after 5 and 30 min of optical breakdown during laser exposure. As a positive control for amyloid fibrils, a solution of lysozyme placed in 3M guanidine was used, which was mixed for 2 or 4 h at 50 °C [32]. It can be seen from the figure that optical breakdown practically does not lead to the formation of amyloid fibrils, although a slight increase in fluorescence is noticeable. 

**Figure 9:**
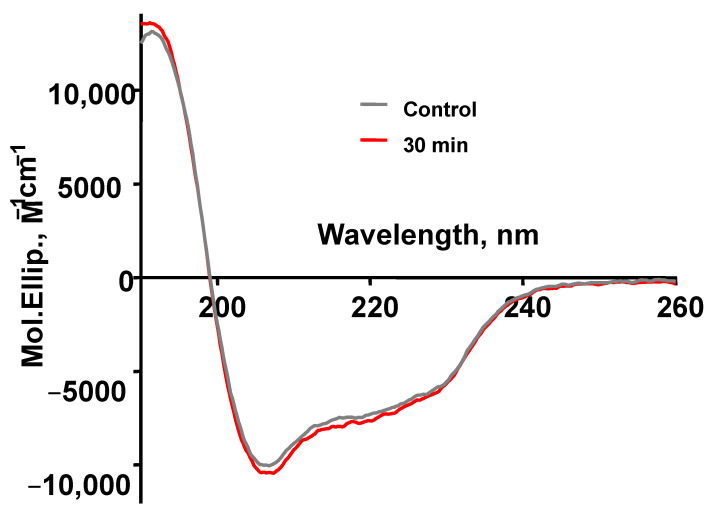
The dispersion curve of circular dichroism control HEWL and after optical breakdowns during laser exposure 30 min.

To see how the structure of the protein molecule changed after optical breakdown, studies of circular dichroism were carried out. Figure 9 and Table 1 show the results of these measurements. It can be seen from the figure that the dispersion curve of circular dichroism after optical breakdown practically coincides with the curve for the control protein. It can be seen from the table that the number of alpha-helices and beta structures after optical breakdown slightly decreased by 1–2%, and at the same time, the number of turns and disordered structures slightly increased.

**Figure 10:**
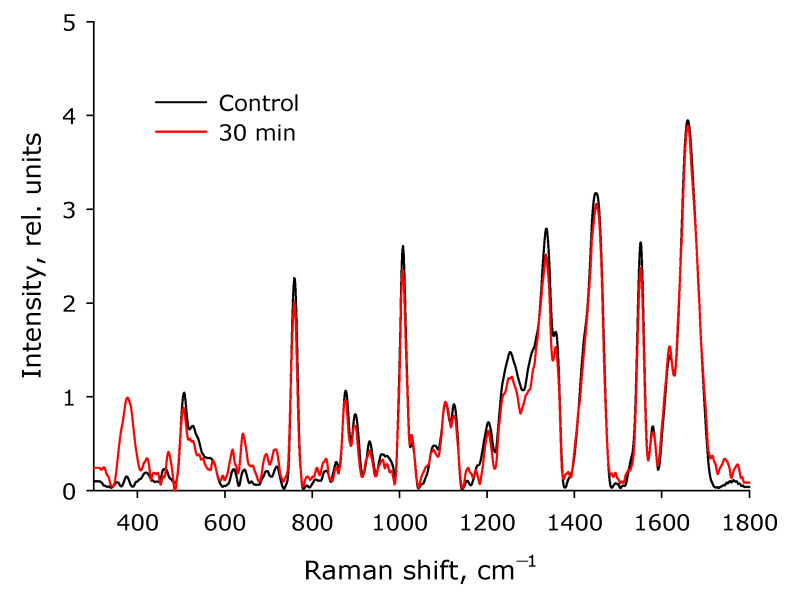
Raman spectra of HEWL for control and after 30 min of laser exposure. The data were obtained using a Raman microscope.

We used Raman spectroscopy to study the structure of the molecule. Raman microscopy was used to study possible changes in the secondary structure of a protein molecule during optical breakdown. The Raman spectra of the native protein and the protein after exposure to laser radiation for 30 min were studied (Figure 10). Even after 30 min of exposure to laser radiation, no significant changes were observed in the protein spectra. The only change recorded in this study was observed in the region of 400 nm. Such a change in the intensity of the Raman signal can only indicate a slight rearrangement within the protein molecule. Thus, we know that structural changes have taken place, but we cannot unambiguously say what they are connected with. To clarify this, the refractive index of the lysozyme solution was measured, and rheological studies were carried out.

**Figure 11:**
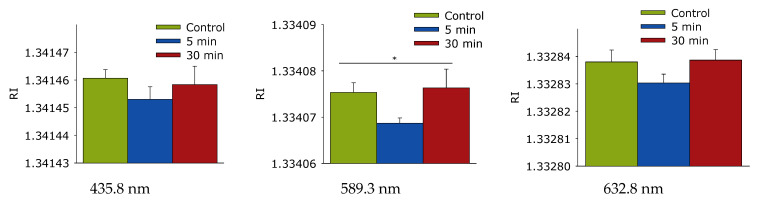
Effect of laser irradiation time on the refractive index of a protein solution of lysozyme at wavelengths of 435.8 nm, 589.3 nm, and 632.8 nm. Data were obtained using precision refractometry (*n* = 3, Mean ± SD). *—*p* < 0.05, significance levels for one-way ANOVA.

The effect of laser irradiation time on the refractive index of a protein solution of lysozyme at wavelengths of 435.8 nm, 589.3 nm, and 632.8 nm was studied (Figure 11). It is shown that the refractive index of lysozyme does not change significantly after exposure to laser radiation for 435.8 and 632.8 nm. Small significant differences were found only for a length of 589.3 nm, with a 5-minute exposure to a decrease in the refractive index.

**Figure 12:**
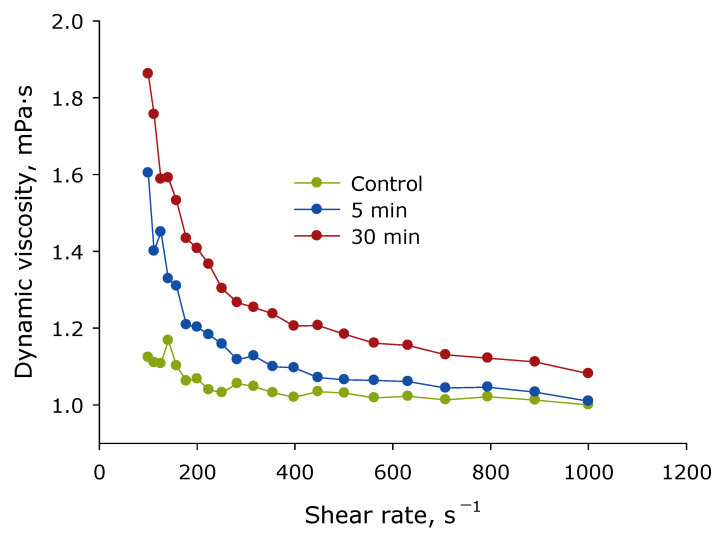
Effect of laser irradiation time on the viscosity of the protein solution of lysozyme at different mixing rates (*n* = 3, Mean).

The effect of laser irradiation time on the viscosity of lysozyme protein solution was studied at different mixing rates (Figure 12). It is shown that the lysozyme molecule’s aqueous solution is characterized by pseudoplasticity. Pseudoplasticity is a property of a fluid characterized by the fact that the viscosity of the fluid decreases with increasing shear stresses. The impact of laser radiation on lysozyme aqueous solutions leads to an increase in viscosity. Moreover, at high mixing rates, the viscosity of the control solution and the solution irradiated for 30 min differ by less than 10%. At low mixing rates, the viscosity of the control solution and the solution irradiated for 30 min differ already by 60%. That is, laser irradiation led to an increase in the pseudoplasticity of the lysozyme solution and an increase in viscosity. That is, the resistance to the movement of one of their parts of the liquid amongst the other increases. That is, protein molecules interact more intensively with each other, usually in protein solutions; this is called aggregation. To verify this assumption, the evolution of the size of light-scattering particles in lysozyme aqueous solution was studied.

**Figure 13:**
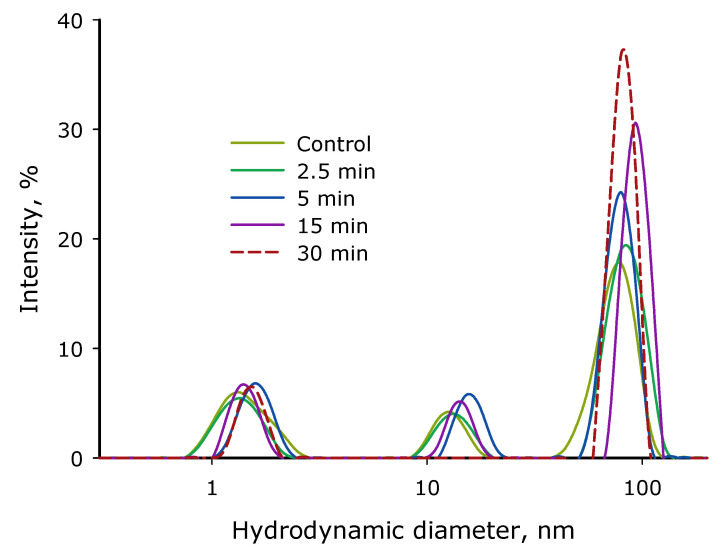
Influence of time of exposure to laser irradiation on the evolution of the size distribution of lysozyme and its aggregates in aqueous solution (*n* = 5, Mean). The data were obtained with the dynamic light scattering method.

The influence of the time of exposure to laser irradiation on the evolution of the size distribution of lysozyme and its aggregates in an aqueous solution was studied (Figure 13). It was shown that the intact preparation contains individual lysozyme molecules, as well as aggregates with an average hydrodynamic diameter of 15 and 80 nm. There are ~4.6 × 10^9^ individual lysozyme molecules per one 80 nm aggregate in solution and ~1.3 × 10^6^ individual lysozyme molecules per one 15 nm aggregate. When exposed to laser radiation, a slight increase in the average hydrodynamic diameter of individual lysozyme molecules is observed. At the time of exposure to laser radiation, an increase in the hydrodynamic diameter of the fraction of individual molecules by almost 30% is seen. It must be said that an increase in the hydrodynamic size of lysozyme aggregates is also observed, and their number also increases. Therefore, after 30 min of exposure to laser radiation, one aggregate of 80 nm in size in solution accounts for 3.1 × 10^9^ individual lysozyme molecules; that is, the number of “large” aggregates increased by a third. In general, the dose-dependent nature of the changes is visible. As the time of laser exposure increases, the changes in the evolution of the size distribution become more and more pronounced.

## 4. Discussion

In the literature, there are practically no data related to the study of optical breakdown for protein conformation in solution. This is largely due to technical difficulties. For the probability of optical breakdown not to decrease, it is necessary to ensure that the laser radiation mode is such that its beam does not cross the site of the last optical breakdown.

In our experiments, the optical breakdown properties weakly depend on the concentration of the HEWL solution. The number of optical breakdowns slightly increases (Figure 2a), and the average luminescence intensity (Figure 2b) decreases by less than a factor of 2 when the protein concentration changes by five orders of magnitude. Characteristically, in a similar study, the changes in these parameters for the optical breakdown in BSA were twice as large [33]. Thus, the size of the protein globule affects the number of optical breakdowns.

The patterns observed when an aqueous solution of lysozyme is irradiated with a laser for different times are extremely similar to the patterns observed in aqueous colloidal solutions of metal nanoparticles [34]. The average distance between optical breakdowns caused by a single laser pulse depends little on the protein concentration (Figure 2c). The average distance between individual breakdowns in a protein solution is much smaller than in a colloidal solution of nanoparticles when exposed to a laser with very similar characteristics (was higher [35]). The average luminescence intensity of optical breakdowns caused by a single laser pulse also has its characteristics. At high concentrations of nanoparticles, when the solution begins to opalize, the average luminescence intensity of optical breakdowns and other breakdown characteristics usually begin to decrease sharply [36], which is not observed in the case of lysozyme protein molecules (Figure 2d). In addition to the effect of the concentration of protein molecules, in this study, the effect of the irradiation time of lysozyme aqueous solution with a laser on the characteristics of the optical breakdown plasma was studied (Figure 3).

The effect of lysozyme concentration on the characteristics of acoustic vibrations induced by optical breakdown was studied (Figure 4). It is shown that the average amplitude of acoustic oscillations induced by optical breakdown does not change significantly at all studied protein concentrations. During the optical breakdown of the aqueous medium, the nanoparticles always show pronounced “concentration” maxima (one or more) that differ significantly in intensity from the “basic” state (as above [37]). At the same time, both with the optical breakdown on individual nanoparticles and with the optical breakdown on protein molecules, the average indicators of acoustic vibrations do not differ significantly [38].

It has been established that after the action of laser radiation on lysozyme aqueous solution, a decrease in lysozyme catalytic activity is observed (Figure 5). A comprehensive analysis of lysozyme protein molecules using optical methods and viscometry was conducted. At the initial stage, it was found that after exposure to laser radiation, an increase in the absorption intensity of protein solutions was observed (Figure 6). Moreover, an increase in optical density is observed, as in the range of absorption of aromatic amino acid residues—this probably indicates their damage—and also in the longer wavelength region (310 nm)—which indicates protein denaturation or aggregation. Thus, several options for the development of events are assumed. During optical breakdown, a large number of both reducing and oxidizing equivalents are formed [39]. There is a generation of ultraviolet radiation, shock acoustic waves, and microvolumes with a significant increase in temperature [40]. With such a set of influences, (1) chemical modification of amino acid residues can be observed; (2) fragmentation of the polypeptide chain; (3) change in the tertiary and secondary structure of lysozyme molecules; (4) partial denaturation; and (5) aggregation of molecules. All of the above scenarios can lead to protein damage and loss of catalytic activity. It should be noted that a similar set of events occurs with proteins under the action of oxidative stress [41], which develops in living systems under the influence of various abiotic factors [42], inflammation [43], hypoxia [44], with the development of many diseases [45,46].

The fluorescence of lysozyme protein solution after exposure to laser radiation was studied (Figure 7). Typically, the maximum excitation occurs in the wavelength range of 275–290 nm [47], and a maximum of 304 nm was registered. This phenomenon is usually observed at high protein concentrations. Under the action of laser radiation, the fluorescence intensity noticeably decreases. In this case, a shift of the emission maximum from 337 nm to 343 nm is observed [48]. Previously, a similar shift of the lysozyme fluorescence maximum was observed during protein denaturation with guanidine hydrochloride. However, fluorescence increased, unlike denaturation with urea.

The chemical modification of the fluorophore usually affects the shape of the emission spot on the 3D fluorescence map. The chemical modification of the fluorophore usually affects the shape of the emission spot on the 3D fluorescence map. In our case, the shape of the fluorescence spot on 3D maps slightly changes after 30 min; a tail appears with a maximum of excitation and emission in the long-wavelength region. However, the shape of the fluorescence of the main spot on 3D maps does not change significantly. Thus, it can be argued that no significant chemical modification of aromatic amino acid residues occurs under the action of optical breakdown. Rather, their degradation occurs, or a significant change in the secondary structure of the molecule may be indicated by the appearance of a tail with a maximum (Ex: 350 nm, Em: 434 nm) at a 30-minute exposure (Figure 7). As already mentioned, the appearance of a new fluorescence peak in this region may be associated with the formation of amyloid fibrils [31]. The appearance of fluorescent peaks in the same wavelength range was also observed for amyloid-like structures of β-amyloid protein [49] and insulin [50].

Aggregation of protein molecules, including “soft”, always leads to a change in rheological properties [51]. It has been established that optical breakdown leads to an increase in the viscosity of lysozyme aqueous solutions and also increases the pseudo-plasticity of the colloidal solution (Figure 12). Such changes in solution indicate a more intensive interaction of protein molecules with each other (aggregation). Interestingly, the viscosity of solutions with amyloid-like structures, on the contrary, can decrease. Thus, in the work with the formation of fibrils from amyloid β, the viscosity of the solution with higher Th-T fluorescence was lower [52].

The main method currently used to assess protein aggregation for the formation of amyloid fibrils is the ThT assay [53]. The ThT assay after laser exposure shows a slight increase in fluorescence (Figure 8); however, this is still well below the levels of the positive control we used [32].

The main feature of aggregation into amyloid-like fibrils is the formation of ordered intermolecular beta-pleated layers [54]. Therefore, it was important to find out how much the secondary structure of the protein changed after laser exposure. For this, we used circular dichroism spectroscopy. It turned out that the curve of circular dichroism in the test sample practically does not differ from the control (Figure 9). However, slight changes (~1–2%) were observed associated with a decrease in alpha helices and beta structures and, at the same time, a comparable increase in turns and disorder structures (Table 1).

Raman microscopy was also used to study possible changes in the secondary structure of the protein molecule during optical breakdown (Figure 10). No significant changes were observed in the spectra of lysozyme; the only change was observed in the region of 400 nm. This change may be related to rearrangement in the β-pleated layer. Changes in the β-pleated layer usually lead to partial denaturation or aggregation of the molecules. With partial denaturation, a significant increase in the number of water molecules in the hydration shell of the protein occurs, while a change in the refractive index is observed [55,56]. The refractive index at different wavelengths was measured with high accuracy (Figure 11) and did not record essential differences.

The study of the size evolution of light-scattering particles in an aqueous solution of lysozyme confirms the development of aggregation (Figure 13). Interestingly, after exposure to laser radiation, there was no shift of the peak of individual molecules to the region of smaller sizes. This indicates the absence of massive damage to the polypeptide chain and the presence of parts of the protein molecule in the solution. The peak of individual molecules, on the contrary, shifts to the region of large sizes (from 1.5 nm to 2 nm at close intensity). An increase in size by 30% in the ball geometry, which is used by the DLS method, corresponds to the formation of dimers of macromolecules. Using formula 4 from [57], it is easy to calculate that there are about two monomers per dimer in the initial state. Thus, it can be argued that most of the lysozyme after 5–15 min of exposure to laser radiation is in the dimer state.

The observed changes in activity, absorption, and fluorescence spectra, in viscosity, as well as in the dynamic light scattering method, indicate a change in the native conformation of the protein. In this case, the changes are not so large as to significantly change the dispersion of circular dichroism, the refractive index of the solution, the Raman spectrum, or significantly increase the fluorescence by the ThT assay. Probably a small part of the protein has degraded, and a part has changed its native state and aggregated, forming functional dimmers or “native-like aggregates” [58]. Similar but less pronounced effects were found for BSA in the paper [33].

## 5. Conclusions

Optical breakdown during laser exposure in protein solutions proceeds with high efficiency: the formation of plasma and acoustic oscillations is observed. At the same time, the physicochemical characteristics of optical breakdown depend rather weakly on the concentration of protein molecules. Changes were observed in the spectra of activity, absorption and fluorescence, and viscosity, as well as in the method of dynamic light scattering. However, the refractive index of the solution and the Raman spectrum showed no significant changes compared to the control. Of particular note is the appearance of a new fluorescence peak (excitation at 350 nm and emission at 434 nm) when the protein solution is irradiated with laser radiation for 30 min. Previously, a peak in this range was associated with the fluorescence of amyloid fibrils. However, neither the ThT assay nor the circular dichroism dispersion confirmed the formation of amyloid fibrils. Probably, a small part of the protein has degraded, and a part has changed its native state and aggregated, forming functional dimmers or “native-like aggregates”.

## Figures and Tables

**Table 1:** Changes in the secondary structure of the protein as measured by circular dichroism in the HEWL control and after optical breakdowns under laser exposure for 30 min.

HEWL	Alpha Helix, %	Beta, %	Turn, %	Disorder, %
Control	31.6	23.4	19.5	25.5
After 30 min optical breakdown	30.6	21.9	20.2	27.3

## Data Availability

Data are available from the authors upon request.
